# Heat-generated meat crust as an intrinsic antioxidant: inhibition of lipid peroxidation and sensory enhancement in food products

**DOI:** 10.1016/j.crfs.2025.101170

**Published:** 2025-08-19

**Authors:** Eylon Asido, Haim Zeigerman, Joseph Kanner, Oren Tirosh

**Affiliations:** Institute of Biochemistry, Food Science and Nutrition, The Robert H Smith Faculty of Agriculture, Food, and Environment, The Hebrew University of Jerusalem, Rehovot, Israel

**Keywords:** Meat Crust, Lipid peroxidation, Meat products, Antioxidants, Enhance sensory preference

## Abstract

Lipid peroxidation is a critical biochemical process that significantly contributes to the deterioration of food quality, particularly in products rich in unsaturated fatty acids. Among the secondary oxidation products, aldehydes such as malondialdehyde (MDA) are responsible for undesirable changes in flavor and nutritional value. Although synthetic antioxidants are commonly employed to mitigate lipid peroxidation, increasing concerns regarding their potential health risks have led the food industry to pursue natural alternatives. In our previous work, we demonstrated that the meat crust formed during cooking has antioxidant properties by inhibiting lipid peroxidation in various systems. The present study aimed to optimize the preparation and yield of meat crust and evaluate its efficacy as a natural antioxidant in preventing lipid peroxidation in soybean oil emulsions and turkey patties. The results indicated that the incorporation of meat crust into turkey patties significantly reduced MDA accumulation. Furthermore, the use of a 9-point hedonic scale grading revealed that patties with added crust were rated more favorably. Additionally, the meat crust demonstrated the ability to remove aldehydic compounds and protect unsaturated fatty acids from oxidative degradation. These findings suggest that meat crust possesses considerable potential as a natural antioxidant, offering an effective strategy to mitigate lipid peroxidation and to improve both the nutritional and sensory appeal of meat products.

## Highlights

• Turkey patties treated with meat crust exhibited lower levels of MDA.
• Panelists preferred turkey patties with meat crust for taste and acceptability.
• Meat crust protected unsaturated fatty acids from oxidation in soybean oil.
• The crust demonstrated the ability to remove aldehydes from samples.
• Meat crust offers a natural alternative to common antioxidants in meat products.

## Graphical abstract

**Figure:**
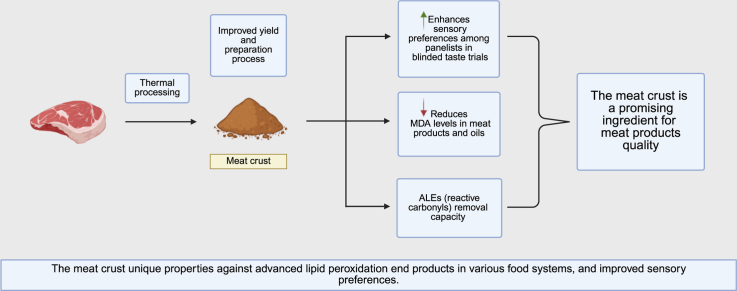

## 1 Introduction

Lipid peroxidation is a complex biochemical process that involves multiple interrelated mechanisms. In its simplest form, unsaturated fatty acids react with molecular oxygen through a free radical-mediated pathway, resulting in the formation of hydroperoxides, which are the primary oxidation products. These hydroperoxides are highly unstable and undergo rapid decomposition, generating a variety of secondary metabolites, including aldehydes. Among these, malondialdehyde (MDA) is frequently used as a biomarker for assessing the extent of lipid peroxidation (Del Rio et al., 2005; Dragoev, 2024; Domínguez et al., 2019).

These compounds at low levels are key degradation products with important implications for food quality, particularly due to their central role in the formation of volatile flavor compounds in meat. However, at higher levels, their impact extends beyond aroma, as the generated aldehydes can interact with proteins, resulting in alterations to both the nutritional value and sensory attributes of the meat (Dragoev, 2024; Domínguez et al., 2019).

Lipid peroxidation is a central process in the deterioration of food quality, particularly in products rich in unsaturated fats. It is a major factor in the onset of rancidity and the degradation of nutritional value. To mitigate lipid peroxidation and preserve food quality, strategies such as reducing oxygen exposure, optimizing storage conditions, and incorporating antioxidants are commonly employed (Aryee et al., 2022; Huang and Ahn, 2019).

Antioxidants such as nitrites, butylated hydroxytoluene (BHT), and butylated hydroxyanisole (BHA) are widely used in the food industry to inhibit lipid peroxidation. However, concerns regarding the safety of these additives have emerged, with several studies suggesting potential associations between prolonged consumption and adverse health effects, including skin allergies, gastrointestinal issues, and an increased risk of cancer (Aryee et al., 2022; Randhawa and Bahna, 2009; Engin et al., 2011; Botterweck et al., 2000; Xu et al., 2021; Manessis et al., 2020; Horbańczuk et al., 2019). As a result, there is a growing consumer demand for alternatives to synthetic antioxidants, presenting a significant challenge for the food industry (Lorenzo et al., 2017).

In our previous work, we demonstrated that the meat crust generated during cooking can reduce lipid peroxidation in various systems (Asido et al., 2024). Our findings indicated that the crust possesses notable reducing activity, marked by the ability to reduce ferric ions to ferrous ions. Specifically, it was shown that beef meat crust can inhibit the oxidation of membrane lipids. In the current study, we further developed the concept of crust as an antioxidant for application in the food industry. We optimized crust preparation and yield, and produced it in powder form, which effectively inhibited the oxidation of soybean oil emulsions and turkey patties. Our findings suggest that the crust may be a promising ingredient for incorporation into food products.

Our aim was to test different crust products as novel antioxidant compounds, to elucidate their capacity to protect oxidizable fatty acids and to scavenge advanced lipid peroxidation end products such as MDA. We compared the effectiveness of crust incorporated into meat patties to common antioxidants in reducing lipid peroxidation. Additionally, we assessed the impact of sensory preference on meat patties containing crust by evaluating overall acceptance and hedonic scores through human sensory testing.

## 2 Materials and methods

### 2.1 Materials

Beef, turkey, and chicken were purchased from local retailers. Ascorbic acid, iron(III) chloride anhydrous, 2-thiobarbituric acid (TBA), butylated hydroxytoluene (BHT), Intralipid 20 % emulsion, Folin-Ciocalteu reagent, methanol, hydrochloric acid (37 %),and catechin were purchased from Sigma-Aldrich (Rehovot, Israel). Chloroform and hexane were purchased from Bio-Lab Ltd. (Jerusalem, Israel). Trichloroacetic acid (TCA) and sodium bicarbonate (SB) were obtained from Merck (Darmstadt, Germany). Sodium dodecyl sulfate (SDS) was purchased from Tivan Biotech (Kefar Sava, Israel). Rosemary extract was generously provided by Lycored Ltd. (Be'er Sheva, Israel).

### 2.2 Crust preparation

5 g of raw beef ribeye steak (entrecote), turkey thigh meat, or chicken breast were used. The average reported fat percentages of the described meats parts are approximately 20 % for beef, 6 % for turkey and 2 % for chicken (U.S. Department of Agriculture, 2019). The meat was flattened to 2.5 mm and placed on a pre-heated stainless-steel pan heated at approximately 155 °C. The meat was flipped 4 times to a total of 4 min of cooking. The crust was ground to a fine powder and was stored at −20 °C. The total yield of the crust standing on ∼ 90 % based on dry weight, compared to the yield utilized previously that stand at only ∼ 8 % (Asido et al., 2024).

### 2.3 Soybean oil emulation and intralipid systems for evaluating lipid peroxidation accumulation

To assess the ability of the crust to attenuate lipid peroxidation, a soybean oil emulsion (SOE) was prepared either by emulsifying commercial soybean oil purchased from local retailers (50 % soybean oil, 0.1 % SDS, and water) or by using Intralipid commercial emulsion. Oxidation system (OS) composed of 100 μM ascorbic acid and 10 μM ferric chloride was added to 1 ml of SOE, and the final volume was adjusted to 5 ml using water. 0.5–2 % w/v of various crust origins were used as antioxidant treatments. Freeze-dried beef (FDB) was also employed as another control when indicated. The FDB was prepared by taking ground beef meat and drying it by lyophilization (DW 6-85-1, Heto-Holten, Denmark) overnight and grinding it to a fine powder. Aliquots from the solutions were collected at specified intervals during incubation for the analysis of lipid peroxidation.

### 2.4 Quantifying lipid peroxidation as malondialdehyde

Lipid peroxidation levels were quantified following the method described by Kanner et al. (Kanner and Harel, 1985), by measuring MDA, a well-established biomarker of lipid peroxidation (Del Rio et al., 2005). Each aliquot was treated with 12 % TCA at a 1:1 ratio and then centrifuged (Heraeus Megafuge 16 R, Thermo Fisher, USA) at 10,000 rpm for 10 min at 4 °C. The resulting supernatant was mixed with 10 mM TBA at a 1:1 ratio, and the samples were incubated in a boiling water bath for 40 min. After incubation, the absorbance was measured at 532 nm (Cary 60 UV–Vis spectrophotometer, Agilent, USA). MDA concentrations were determined using the conversion factor of 1 μmol/L = 0.156 absorbance and were expressed as MDA equivalents (nmol/g of meat, or nmol/ml of emulsion).

### 2.5 Comparison of the antioxidant's ability of rosemary extract, catechin and meat crust

#### 2.5.1 Quantification of polyphenols in rosemary extract

The quantification of polyphenols in the extract was performed using the Folin-Ciocalteu method, as described by Singleton et al. (Singleton and Rossi, 1965). Briefly, 2.5 ml of commercial rosemary extract was dissolved in 10 ml of hexane: water: methanol mixture (50:30:20). Following thorough mixing and centrifugation at 3500 rpm for 10 min at 4 °C, 100 μl of the polar phase was transferred to 500 μl of diluted Folin-Ciocalteu reagent (1:10) (47641-100 ML-F). The resulting mixture was shaken for 5 min and subsequently supplemented with 400 μl of 7.5 % SB solution, and the resulting mixture was incubated at room temperature for 30 min. After the incubation, the absorbance of the mixture was measured at 765 nm, and the results were compared to a standard curve prepared using catechin as the reference.

#### 2.5.2 Comparing lipid peroxidation accumulation in turkey patties treated with rosemary extract, catechin, and beef meat crust

Rosemary extract polyphenol concentration used in the turkey patty was 24–240 μM. Catechin concentration used was also 24–240 μM, and beef meat crust was incorporated into turkey patties at 0.2–2 % w/w. All treated patties were compared to the control with no additives. Patties were formulated with 5 % water and 1.2 % salt. The patties were cooked using a Ninja Grill (A6551EU, SharkNinja, UK) for a total of 8 min (4 min per side) at 250 °C. After cooking, the patties were stored at −80 °C. The patties were taken directly from −80 °C and reheated in a microwave (WP800AL20, Gold Line, Israel) for 30 sec. Subsequently, 1 g from each patty was homogenized (Polytron PT 3000) with 3 ml of water and followed by MDA quantification as mentioned in Section 2.4.

#### 2.5.3 Evaluating rosemary extracts and catechin ability to reduce lipid peroxidation versus beef meat crust in soybean oil emulsion with oxidation system

To evaluate the efficacy of the meat crust in reducing lipid peroxidation levels relative to rosemary extract and catechin, a SOE prepared from commercial soybean oil was used in combination with the OS described in Section 2.3. The meat crust was administered at a concentration of 100 mg (2 % w/v), while rosemary extract concentrations ranged from 0.06 to 0.96 mM, and catechin concentrations ranged from 0.4 to 1.6 mM. Following 60 min of incubation at 37 °C, MDA levels were quantified using the method outlined in Section 2.4.

### 2.6 Hedonic changes and oxidation analysis in turkey patties

To evaluate the potential of the beef crust to enhance the overall acceptance of cooked turkey patties, blinded taste trials were conducted. Twenty-four participants (seven females and seventeen males), ranging in age from 20 to 80 years, were recruited. All participants provided informed consent prior to participation. Each participant received two 20 g turkey patties, one of which contained 2 % w/w crust incorporated. All patties were prepared as described in Section 2.6.2. Participants evaluated the patties using a 9-point hedonic scale which is a widely used method for assessing consumer liking in food science (Lawless et al., 2010). Participants were encouraged to provide additional comments. Additionally, to assess the effect of the crust on lipid peroxidation in relation to sensory perception, participants were asked to cut approximately one-third of each patty for subsequent analysis of MDA levels (Section 2.5.2).

### 2.7 Evaluating the crust protective effect on fatty acids profile

Total lipids were extracted from the samples using the Folch method (Sündermann et al., 2016). The SOE from intralipid was utilized follow by applying the OS system. Meat crust was added at a concentration of 2 % w/v, and the tubes were incubated for 16 h at 37 °C. The samples were then immediately treated with 12 % TCA at a 1:1 ratio and centrifuged at 10,000 rpm for 10 min at 4 °C. An aliquot of the resulting supernatant was mixed with four volumes of a chloroform-methanol (2:1 v/v) solution, and the lower phase was collected following centrifugation at 5000 rpm for 10 min at 4 °C. The lipid extracts were evaporated using a SpeedVac (Concentrator vacufuge 5301, Eppendorf, Germany) for 60 min at room temperature. The dried lipids were then converted into fatty acid methyl esters (FAMEs) through trans-methylation using methanol containing 1 % HCl. FAMEs were extracted from the reaction mixture using hexane. Gas chromatography-flame ionization detection (GC-FID) analysis of FAMEs was performed using a gas chromatograph equipped with a flame ionization detector (Model 7890A, Agilent, USA). FAMEs were separated on a DB-23 capillary column (60 m × 0.25 mm × 0.25 μm, Agilent, USA), with hydrogen used as the carrier gas. The analysis was conducted by the Interdepartmental Analytical Unit (TSABAM) at the Faculty of Agriculture, Food, and Environment, Rehovot.

### 2.8 Statistical analysis

Statistical analyses were performed with JMP Pro 18.0.0 (SAS Institute, Inc.) software. The results were expressed as means ± standard deviation (SD). The data were analyzed using analysis of variance, with a one-way ANOVA followed by a post hoc Tukey-Kramer HSD test, Student's t-test, or paired t-test. Differences were considered significant at *p ≤ 0.05* and were indicated by different letters or by asterisks.

## 3 Results

### 3.1 Stability of the crust after storage

**Fig. 1:**
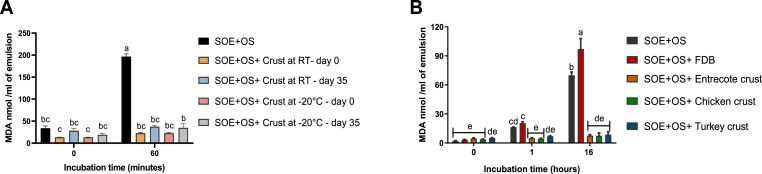
(A) Stability of the beef meat crust during 35 days of storage at room temperature (RT) or −20 °C. The antioxidant stability of the crust (25 mg) was assessed by measuring MDA accumulation in a SOE prepared from commercial soybean oil, using an OS containing ascorbic acid (100 μM) and ferric chloride (10 μM). After 60 min of incubation at 37 °C, MDA levels were quantified. (B) Lipid peroxidation-reducing capacity of meat crusts from different sources. The ability of meat crusts (100 mg each) derived from various animal sources to inhibit lipid peroxidation was evaluated. FDB (100 mg) was included as an additional control. The SOE used in this assay was prepared from an intralipid solution and combined with the OS. MDA levels were measured immediately, after 1 and 16 h of incubation at 37 °C. Statistical analysis was performed using the Tukey-Kramer HSD test (n = 3), and differences were considered statistically significant at p ≤ 0.05, as indicated by different letters.

The antioxidant properties of the beef crust remained robust, with its capacity to reduce MDA levels remaining stable after 35 days of storage. Notably, storage temperature did not significantly affect the crust's antioxidative activity, and the crust maintained its efficacy in preventing the oxidation of SOE during a 60 min incubation at 37°C, compared to untreated SOE (*p < 0.0001*) (Fig. 1A).

### 3.2 Different types of meat crust reduce MDA levels in SOE

Crusts derived from beef, turkey, or chicken effectively inhibited the increase in MDA levels compared to the unprotected control. The crust protected SOE for up to 16 h of incubation at 37 °C (*p < 0.0001*). In contrast, the freeze-dried beef powder exhibited a pro-oxidant effect, as evidenced by significantly higher lipid peroxidation levels compared to those observed in untreated SOE (*p < 0.0001*) (Fig. 1B).

### 3.3 Antioxidant activity of rosemary extract, catechin, and beef meat crust in turkey patties

Turkey patties treated with 0.2 % meat crust exhibited lower lipid peroxidation levels than untreated control samples (*p = 0.0001*). Similarly, the incorporation of 24 μM catechin resulted in a comparable reduction in MDA content to that observed in patties treated with 0.2 % crust. Notably, the addition of 2 % meat crust led to a more pronounced decrease in MDA levels than either the 0.2 % crust or 24 μM catechin treatments (*p = 0.0063* and *p = 0.0085*, respectively).

**Fig. 2:**
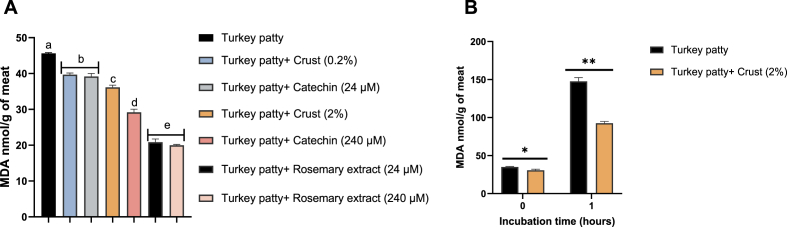
The effect of beef meat crust on reducing MDA levels in turkey patties. (A) Turkey patties were treated with rosemary extract (24–240 μM), catechin (24–240 μM), and meat crust (0.2–2 % w/w). The patties were cooked using a Ninja Grill and, after cooking, stored at −80 °C until use. Prior to analysis, patties were reheated in a microwave and assessed for their MDA concentrations. (B) Turkey patties supplemented with 2 % w/w beef meat crust were compared to untreated patties. Both sets of patties were homogenized, and MDA levels were measured immediately after homogenization and after 1 h of incubation at 37 °C. Statistical analysis was performed using the Tukey-Kramer HSD test for panel A and the Student's t-test for panel B (n = 3). Differences were considered significant at p ≤ 0.05 and are indicated by different letters or asterisks (∗p ≤ 0.05, ∗∗p ≤ 0.0001).

However, a higher catechin concentration (240 μM) resulted in significantly lower MDA levels compared to the crust treatments (*p < 0.0001*). Both concentrations of rosemary extract produced similar reductions in lipid peroxidation (Fig. 2A).

To further assess the antioxidant activity of the crust after cooking, we incubated the patties at 37 °C for 1 h. Surprisingly, the 2 % crust retained its antioxidative efficacy and significantly reduced lipid peroxidation in turkey patties compared to the untreated control (*p = 0.0001*) (Fig. 2B).

### 3.4 Favorable hedonic rating and anti-lipid peroxidation activity of meat crust in turkey patties

**Fig. 3:**
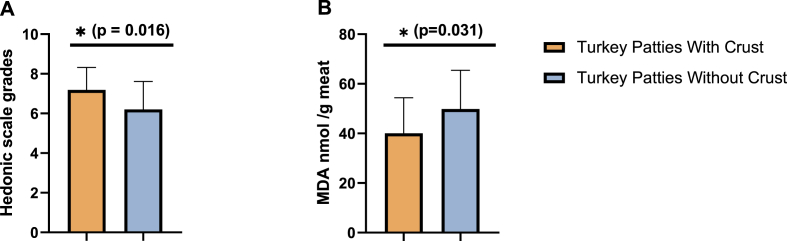
The beef meat crust capacity at decreasing lipid proxidation levels in turkey patties with favorable hedonic rating. Twenty-four participants were provided with two turkey patties, one of which had 2 % w/w meat crust incorporated. The participants rated the patties using a 9-point hedonic scale and samples collected from all patties for MDA levels. (A) Participants' average hedonic scale grades of turkey patties with and without crust (B) MDA levels of turkey patties with and without crust. The statistical analysis was performed using Paired t-Test for panel A (n = 24) and Student's t-test for panel B (n = 24 patties with crust and n = 24 patties without crust. Each patty was analyzed in triplicate). Differences were considered significant at p ≤ 0.05 and are indicated by asterisks.

Turkey patties formulated with 2 % w/w beef meat crust were found more favorable with a significant difference in preference by panelists as determined by blinded taste evaluation trials.(Fig. 3A). Notably, the patties supplemented with meat crust also had significantly reduced lipid peroxidation levels, as indicated by lower MDA accumulation relative to untreated controls (Fig. 3B).

### 3.5 Neutralizing capacity of reactive carbonyls (aldehydes) by beef crust

Soybean oil emulsion was incubated and oxidized using an OS containing ascorbic acid and iron chloride for 16 h. Following oxidation, 100 mg of meat crust or FDB, with or without BHT (0.1 %), was added to the oxidized oil. MDA levels following treatment with the meat crust were significantly reduced after just 1 h of incubation compared to the SOE subjected to OS alone (*p = 0.0012*). Moreover, after 5 h of incubation, crust treatment further decreased MDA concentrations (*p < 0.0001*).

**Fig. 4:**
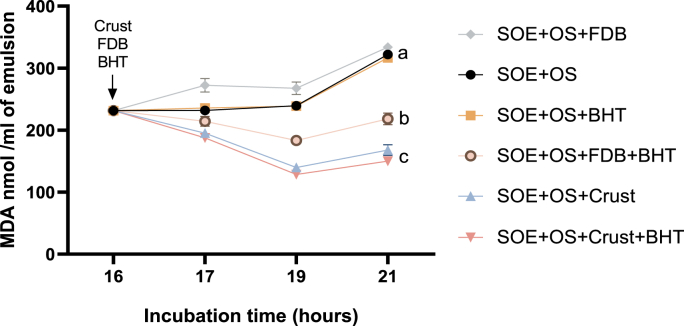
Meat crust ability to remove reactive carbonyls. SOE was prepared from intralipid in combination with the OS. After 16 h of incubation at 37 °C, either BHT (0.1 %) or 100 mg of beef meat crust or FDB, with or without BHT, were added. The samples were then incubated for an additional 5 h, for a total incubation time of 21 h. At designated time points, aliquots were collected to quantify MDA accumulation. Statistical analysis was performed on MDA levels after 21 h of incubation using the Tukey-Kramer HSD test (n = 3). Differences were considered significant at p ≤ 0.05 and are indicated by different letters.

In contrast, FDB, included as an additional control, exhibited a pro-oxidant effect with a significant increase in MDA levels after 5 h of incubation compared to the crust-treated samples (*p < 0.0001*). However, the inclusion of BHT in samples containing FDB mitigated lipid peroxidation, as indicated by reduced MDA levels relative to FDB-treated samples without BHT (*p < 0.0001*) (Fig. 4).

### 3.6 Antioxidant protection of unsaturated fatty acids by crust treatment

**Fig. 5:**
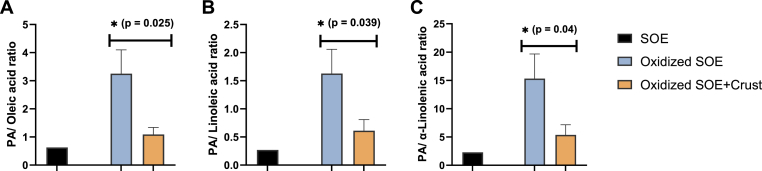
Protective effect of the beef meat crust on unsaturated fatty acids in SOE. SOE was prepared from intralipid and was treated with an OS. Meat crust (100 mg) was added to one of the samples. After 16 h of incubation at 37 °C, total lipids were extracted and converted into fatty acid methyl esters (FAMEs). The ratios of PA to oleic, linoleic, and α-linolenic acids were calculated and compared to those in unoxidized intralipid SOE. Statistical analysis was performed on the PA-to-unsaturated fatty acid ratios, with and without crust treatment, using the Student's t-test (n = 3). Differences were considered significant at p ≤ 0.05 and are indicated by asterisks.

The protective effect of the beef crust on the fatty acid composition of SOE, originating from an intralipid solution, was assessed following a 16 h incubation with the oxidative system. The results are expressed as the ratio of palmitic acid (PA) to unsaturated fatty acids, relative to untreated, non-incubated SOE. Treatment with the meat crust resulted in a marked preservation of oleic, linoleic, and α-linolenic acids compared to untreated SOE, indicating an early phase protective effect against oxidative degradation of fatty acids (Fig. 5A, B, and 5C, respectively).

### 3.7 Antioxidant effect of meat crust, rosemary extract, and catechin in SOE

**Fig. 6:**
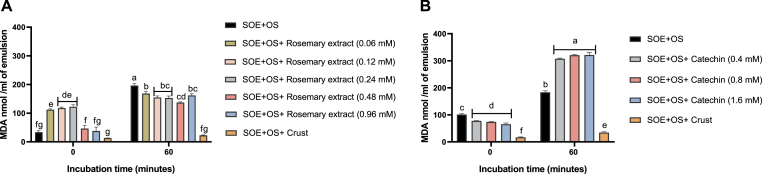
Comparison of the antioxidant activity of meat crust from beef with rosemary extract and catechin in SOE. An SOE prepared from commercial soybean oil was used in combination with the OS. Meat crust (100 mg) was added in both panels A and B. In panel A, rosemary extract was tested at polyphenol concentrations ranging from 0.06 to 0.96 mM. In panel B, catechin was tested at concentrations ranging from 0.4 to 1.6 mM. Following 60 min of incubation at 37 °C, MDA levels were quantified. Statistical analysis was performed using the Tukey-Kramer HSD test (n = 3), and differences were considered significant at p ≤ 0.05, as indicated by different letters.

The antioxidant capacity of the meat crust originating from beef was evaluated in comparison to rosemary extract and the antioxidant polyphenol catechin, both tested at various concentrations. The meat crust demonstrated a significantly greater ability to reduce MDA levels after 60 min of incubation, relative to both rosemary extract and catechin, regardless of the concentrations applied (Fig. 6A and B, respectively).

## 4 Discussion

Previously, we showed that the natural meat crust formed during cooking exhibits reducing activity and can inhibit the oxidation of lipids in meat (Asido et al., 2024). To further explore the potential of meat crust as a functional ingredient, we produced crust powder from different meat sources with high yield and compared its antioxidant properties to known antioxidants in various oxidizable food systems.

Incorporation of 2 % meat crust into turkey patties resulted in significantly lower MDA levels compared to both 24 μM catechin and untreated controls. One clear advantage of the crust over rosemary extract is its lack of a strong, characteristic aroma; the aroma of rosemary was prominent even at low concentrations and likely altered the organoleptic properties of the patties. Furthermore, challenges associated with natural antioxidants, such as sensory impact and formulation compatibility, remain to be addressed.

Natural plant-based antioxidants, while effective, often exhibit low stability, potentially compromising the shelf life of meat products. Additionally, their extraction and incorporation can increase production costs, affecting the economic feasibility of widespread use in the food industry (Manessis et al., 2020; Barbosa et al., 2023; Oswell et al., 2018). In this context, the meat crust presents a promising ingredient. The use of meat crust can offer significant cost benefits for industrial applications. In the final product, traditional meat is replaced with a modified meat crust, and can be compensated for water loss during the crust preparation process. Additionally, a less expensive meat mixture can be utilized for creating the crust. Ultimately, the economic feasibility will depend on the scalability of the process. As demonstrated in our study, the crust remains stable for up to 35 days regardless of storage temperature, and its preparation is both rapid and efficient. The crust's stability is probably inherited from its high heat preparation technique. These features offer distinct advantages over conventional natural antioxidants such as rosemary extract.

To further evaluate the potential of meat crust as an integrated antioxidant ingredient, hedonic ratings assessments were conducted comparing turkey patties supplemented with beef crust to untreated controls. The results demonstrated that patties containing meat crust were rated more favorably by participants. Previous studies support the notion that the formation of a flavorful crust during cooking enhances the overall taste and aroma of meat products (Kilic et al., 2023; Yoo et al., 2020). In addition to improved sensory acceptability, crust-treated patties exhibited significantly lower levels of lipid peroxidation compared to untreated samples. These findings underscore the dual functionality of meat crust as both an effective antioxidant and a contributor to improved customers preferences in meat products. Although, the current sensory evaluation involved 24 participants and solely hedonic scale was employed, the results indicate significant preference for crust-containing patties among panelists. However, further validation using comprehensive sensory profiling and larger, demographically diverse using professional panelists is warranted to validate these results.

In our previous study, we demonstrated that the meat crust possesses reductive properties capable of converting ferric ions (Fe^3+^) to ferrous ions (Fe^2+^). To further explore its mechanisms of action, we investigated the ability of meat crust to reduce aldehydes (reactive carbonyls) present in oxidized SOE. Treatments involving meat crust showed a significant unique effect reduction in aldehyde concentrations in the oxidized SOE samples. Although the precise mechanisms underlying this aldehyde-reducing activity remain to be elucidated and further analysis is needed to identify the specific bioactive compounds, these findings suggest that the presence of meat crust during digestion may help reduce absorbed aldehydes in the gastrointestinal tract.

These results are particularly noteworthy, as dietary advanced lipid oxidation end products (ALEs) are increasingly recognized as contributors to various health issues, including cognitive decline and cardiovascular diseases (Grootveld et al., 2020; Wang et al., 2024). Therefore, incorporating meat crust may serve as a practical strategy to minimize ALEs formation and reduce their absorption. The FDB, when combined with high levels of chain-breaking antioxidants like BHT, had some capacity to remove aldehydes, although it is significantly less effective than the crust. FDB acts as a prooxidant, likely due to its oxidizability potential, which includes iron, reducing compounds, and polyunsaturated fatty acids (PUFAs). An evaluation of the initial rate of aldehyde removal by FDB in SOE suggests a pro-aldehyde formation effect. However, this effect can be inhibited by BHT until the BHT is depleted. In contrast, the crust functions as an antioxidant from the outset, as it begins to eliminate aldehydes immediately upon being added to the oxidized emulsion.

While the meat crust has been shown to reduce MDA levels in both SOE and meat patties, it was also important to demonstrate preservation of PUFAs in these food systems. Therefore, we investigated the effects of the crust on the fatty acid profile in SOE. Our results confirmed that the crust effectively prevented the initial phase of lipid peroxidation and preserved α-linolenic acid, and linoleic acid levels compared to oxidation without crust. Additionally, the crust demonstrated protective effects against the oxidative degradation of oleic acid, indicating protection against reactive oxygen species (ROS) that directly compromise the single double bond in this molecule, such as singlet oxygen (Shimizu et al., 2019).

To assess whether the protective properties of the meat crust are specific to beef, we prepared crusts from turkey and chicken meat and, using the SOE with the oxidation system, compared the ability of these crusts to prevent MDA accumulation relative to untreated samples. The results demonstrated that all types of meat with various fats percentages (U.S. Department of Agriculture, 2019) effectively inhibited lipid peroxidation, with no significant differences observed between them. These findings indicate that low-cost meat crust from various sources can serve as effective antioxidants.

In addition to the comparative analysis conducted with meat crust in turkey patties, we employed the SOE system combined with the oxidation system as a complementary model to further evaluate the antioxidant efficacy of meat crust relative to rosemary extract and catechin. In this model, meat crust exhibited a pronounced protective effect by completely preventing the increase in MDA levels following incubation, thereby outperforming all tested concentrations of both rosemary extract and catechin.

Although polyphenols were also effective antioxidants in the turkey patty model, these differences may be attributed to the distinct mechanisms of action and interactions between polyphenolic compounds and myoglobin, which may facilitate their antioxidant activity (Rabkin et al., 2022; Lapidot et al., 2005). However, in pure lipid systems, the crust was highly effective and exhibited antioxidant effects with no pro-oxidant properties.

We also acknowledge the concern regarding potential heterocyclic amine (HCA) formation during meat crust preparation. HCAs are mutagenic and potentially carcinogenic compounds that can form when meat is cooked at high temperatures for extended periods (Skog, 2009). Our cooking protocol—preheating a stainless-steel pan to approximately 155 °C and cooking for 4 min with four flips—was designed to maximize crust yield while minimizing excessive surface heating and cooking time, thereby reducing HCA formation. Previous research has shown that cooking meat at temperatures ≤150 °C yields negligible levels of HCAs (Skog et al., 1997), and that substantial HCA formation generally requires temperatures ≥175–200 °C and prolonged heating (Gibis and Weiss, 2016). Under our cooking conditions, the short cooking time and frequent flipping limited localized overheating and charring, further mitigating HCA production. Moreover, Felton et al. demonstrated that cooking meat at approximately 160 °C with frequent turning not only ensured microbial safety but also produced low or undetectable HCA levels (Felton and Knize, 1994). Nevertheless, we recognize that a full toxicological assessment of the crust powder is needed.

## 5 Conclusion

While commonly used natural antioxidants, such as rosemary extract, offer health benefits and align with consumer preferences, additional innovative strategies such as utilizing a safe heat treatments that facilitate the appearance of antioxidant properties in organic metrics, are a promising direction. The protective capacity of meat crust, which offers dual functionality as an antioxidant through multiple mechanisms with a clear consumer's hedonic preference toward crust-containing products, could be an important alternative for the meat industry.

## CRediT authorship contribution statement

Eylon asido: Conceptualization, Methodology, Formal analysis, Investigation, Writing- original draft Project administration. Haim Zeigerman: Methodology, Formal analysis, Writing-review & editing. Joseph Kanner: Conceptualization, Validation, Writing-review & editing, Project administration. Oren Tirosh: Conceptualization, Methodology, Validation Investigation, Resources, Writing-review & editing, Supervision, Project administration, Funding acquisition.

All authors have read and agreed to the published version of the manuscript.

## Funding

This research did not receive any specific grant from funding agencies in the public, commercial, or not-for-profit sectors.

## Declaration of competing interest

The authors declare that they have no known competing financial interests or personal relationships that could have appeared to influence the work reported in this paper.

## Data Availability

Data will be made available on request.
